# Sodium-Glucose Cotransporter Type 2 Inhibitors Use in Elderly Polypathological Patients with Acute Heart Failure: PROFUND-IC Registry

**DOI:** 10.3390/jcm13123485

**Published:** 2024-06-14

**Authors:** Alicia Guzmán-Carreras, Andrea María Vellisca-González, Juan Igor Molina-Puente, Rocío García-Alonso, Mateo Paz-Cabezas, Beatriz Sánchez-Sauce, Fernando Aguilar-Rodríguez, María Del Rosario Iguarán-Bermúdez, Emmanuel Andrès, Noel Lorenzo-Villalba, Manuel Méndez-Bailón

**Affiliations:** 1 Servicio de Medicina Interna, Hospital Clínico San Carlos, Facultad de Medicina, Universidad Complutense de Madrid, Instituto de Investigación Sanitaria Hospital Clínico San Carlos (IdISSC), 28040 Madrid, Spain; 2 Servicio de Medicina Interna, Complejo Asistencial de Ávila, 05071 Ávila, Spain; 3 Unidad de Apoyo Metodológico a la Investigación, Servicio de Medicina Preventiva, Hospital Clínico San Carlos, Instituto de Investigación Sanitaria Hospital Clínico San Carlos (IdISSC), 28040 Madrid, Spain; 4 Servicio de Medicina Interna, Fundación Hospital de Alcorcón, 28922 Madrid, Spain; 5 Servicio de Medicina Interna, Hospital 12 de Octubre, 28041 Madrid, Spain; fernando.aguilar.rodriguez@gmail.com; 6 Service de Médecine Interne, Hôpitaux Universitaires de Strasbourg, 67200 Strasbourg, France

**Keywords:** sodium-glucose co-transporter-2 (SGLT2) inhibitors, elderly, heart failure

## Abstract

**Background/Objectives:** Heart failure (HF) is a highly prevalent clinical syndrome with serious morbidity and mortality. Furthermore, acute heart failure (AHF) is the main cause of hospital admission in people aged 65 years or more. Sodium-glucose cotransporter type 2 inhibitors (SGLT2is) have been shown to improve the survival and quality of life in patients with HF regardless of left ventricular ejection fraction (LVEF). Our aims were to describe the characteristics of adults with multiple pathologies admitted with acute heart failure as the main diagnosis and of the population treated with SGLT2is, as well as to evaluate if their use was associated with lower readmission and mortality rates. **Methods**: A prospective study of patients from the PROFUND-IC registry who were admitted with AHF as the main diagnosis was conducted. Clinical and analytical characteristics were analyzed, as well as readmissions and mortality. Descriptive and bivariate analyses of the sample between those taking SGLT2is and those who were not were performed, using the chi-square test for qualitative variables and Welch’s test for quantitative measures, as well as the Fisher and Wilcoxon tests as indicated for nonparametric tests. Kaplan–Meier curves were constructed to analyze the readmission and mortality of patients at 12 months based on SGLT2i treatment. Finally, a propensity score matching was performed, guaranteeing that the observed effect of the drug was not influenced by the differences in the characteristics between the groups. **Results:** There were 750 patients included: 58% were women, and the mean age was 84 years. Functional class II according to the NYHA scale predominated (54%), and the mean LVEF was 51%. SGLT2 inhibitors were prescribed to only 28% of patients. Most of the patients were men (48.6% vs. 39.8%, *p* = 0.029), they were younger (82 vs. 84 years, *p* = 0.002), and their LVEF was lower (48% vs. 52%, *p* < 0.001). Lower mortality was observed in the group treated with SGLT2is, both during baseline admission (2.4% vs. 6.9%, *p* = 0.017) and at the 12-month follow-up (6.2% vs. 13%, *p* = 0.023); as well as a lower readmission rate (23.8% vs. 38.9%, *p* < 0.001). After the propensity score matching, a decrease in the 12-month readmission rate continued to be observed in the group treated with SGLT2is (*p* = 0.03). **Conclusions:** SGLT2is use was associated with lower readmission rates at the 12-month follow-up in older adults with multiple pathologies admitted with acute heart failure.

## 1. Introduction

Heart failure (HF) is a highly prevalent clinical syndrome, affecting 65 million people worldwide, and its prevalence will continue to increase in the upcoming years because of the aging of the population and improved outcomes regarding survival of the disease. In addition, this syndrome is associated with significant morbidity and mortality, loss of quality of life, and an increased number of hospital admissions, particularly in older adults [1,2]. 

The prevalence of HF in Spain varies from 2 to 5% [3,4,5], depending on the series, and prevalence increases with age, reaching 9% in octogenarians [4]. Moreover, acute heart failure is the main cause of hospital admission in people aged 65 years or more [5]. Half of these patients present with a preserved left ventricular ejection fraction (LVEF), a syndrome that represents a diagnostic and therapeutic challenge, partially due to its unknown pathophysiology [2].

Sodium-glucose cotransporter type 2 inhibitors (SGLT2is) are natriuretic and glycosuric drugs that have been demonstrated to improve the quality of life and survival of patients with HF, regardless of LVEF and the presence of diabetes. They also decrease renal function impairment [6]. The European and American guidelines on HF recommend including SGLT2is in the treatment of heart failure with reduced ejection fraction [7,8]. In addition, the latest available evidence proves their ability to reduce the combined end point of hospital admission for HF and cardiovascular death in patients with preserved left ventricular ejection fraction (HFpEF) [9,10]. Therefore, SGLT2is are also currently recommended in this group of patients according to the latest update of the guidelines [11,12].

Patients with multiple pathologies represent a therapeutic challenge regardless of age. The Charlson comorbidity index (CCI) defines patients as polypathological when they have a score equal to or greater than three. A high CCI is associated with increased readmissions in patients with heart failure [13]. Furthermore, comorbidities and their associated polypharmacy make treatment optimization difficult due to the risk of interactions, readmissions, and mortality. In a recent study of those with severe heart failure with reduced ejection fraction, the prescription and up-titration of guideline-directed medical therapy remained limited [14].

There is still caution about starting treatment for chronic heart failure (CHF) during an episode of AHF. The STRONG-HF study analyzed the optimal time to start the guideline-directed medication of HF including renin-angiotensin blockers (ACEIs/ARBs), beta-blockers (BBs), and mineralocorticoid receptors antagonists (MRAs), although SGLT2is were not included [15]. Several studies have also supported the use of acetazolamide and thiazides combined with furosemide in acute heart failure to achieve the sequential blockade of nephron [16,17]. Regarding SGLT2is, the EMPULSE clinical trial [18] and the DICTATE-AHF [19] study have explored the use of empagliflozin and dapagliflozin, respectively, in AHF. 

The aim of this study was to describe the characteristics of patients with multiple pathologies admitted for acute heart failure regarding the prescription of SGLT2is and their differential characteristics, as well as the impact of these drugs on readmission and mortality at the 12-month follow-up. 

## 2. Methods

A prospective observational cohort study including 750 patients with multiple pathologies admitted to Internal Medicine with AHF as their main diagnosis was carried out. Data were collected from the PROFUND-IC multicenter registry in the period between October 2020 and May 2023. The PROFUND-IC registry is a project developed within the framework of the study groups on Heart Failure and Polypathological Patients and Advanced Age of the Spanish Society of Internal Medicine. Up to 32 hospitals have been collaborating on the registry, incorporating patients according to standard inclusion and exclusion criteria. Inclusion criteria included acute heart failure (AHF) as the primary diagnosis and NT-proBNP more than >1500 pg/mL upon admission, patient with polypathologies (defined as 2 or more associated chronic diseases) and signed written consent. AHF denotes both de novo (first episode of AHF) and chronic decompensated heart failure. The only exclusion criterion was the lack of information on SGLT2is prescription at hospital discharge.

The PROFUND-IC registry was created before SGLT2is agents became available for heart failure. We selected all patients who took these medications in order to determine whether they benefited from them. The reason for the prescription could be HF or another indication, so patients before the publication of the guidelines could have been treated with these drugs. 

Baseline epidemiological variables such as age, sex, weight, height, and past medical history (diabetes mellitus, dyslipidemia, arterial hypertension, chronic kidney disease, atrial fibrillation, ischemic heart disease, chronic respiratory diseases, cerebrovascular disease, cognitive impairment, peripheral arterial disease, and neoplasia) were collected. We also collected results on the Barthel scale, Short Physical Performance Battery (SPPB), and PROFUND index. Clinical variables such as HF etiology, functional class according to the New York Heart Association (NYHA), and LVEF as well as analytical variables (NT-proBNP, CA-125, creatinine, and glomerular filtration rate according to CKD-EPI) were also recorded. Prognostic variables were included, such as the risk of mortality at 30 days according to the multiple estimation of risk based on the emergency department Spanish score in patients with AHF (MEESI-AHF) scale, and prediction of mortality at one and three years using Meta-analysis Global Group in Chronic Heart Failure (MAGGIC) scale. Treatment at the time of discharge was also registered. During the 12-month follow-up period, we recorded the mortality rate (both during admission and at the 12-month follow-up), particularly whether it was due to cardiovascular causes, and readmissions. 

Quantitative variables are expressed as mean and standard deviation (or median and interquartile range if they were not adjusted to normality), and qualitative variables are presented as absolute values and percentages. A descriptive analysis was carried out, as well as a bivariate analysis comparing the characteristics of the patients regarding SGLT2is treatment. Association with qualitative variables was analyzed using Pearson’s chi-squared test (or Fisher’s exact test, when required). Regarding the quantitative measures, the Welch two-sample test was performed with variables following a normal distribution and the Wilcoxon rank sum test in those requiring a nonparametric test. Kaplan–Meier curves were constructed analyzing the readmission and mortality of patients, comparing the two groups based on SGLT2is treatment using a log-rank test. A univariable Cox proportional hazards model was used to determine the hazard ratio associated with the variable being studied. The level of statistical significance was set at *p* < 0.05. 

Finally, propensity score matching was carried out to evaluate if the effect of SGLT2is was due to the treatment itself or due to the differences in the characteristics between the groups. Variables that impacted in the severity of AHF were included (sex, age, systolic blood pressure (SBP), LVEF, creatinine, NT-proBNP, diabetes mellitus, and chronic kidney disease). The distribution of SBP, creatinine, and chronic kidney disease was homogeneous in both groups, so they were excluded from the analysis to avoid reducing the effectiveness of the test. Furthermore, only patients with LVEF > 40% were included in this analysis to ensure that the observed differences were due to the use of SGLT2is and in order to avoid interactions with the rest of the treatments with demonstrated efficacy in HF with reduced ejection fraction. Moreover, to ensure the observed effect was attributed exclusively to the SGLT2is, the treatments in which statistically significant differences were observed in their administration between the two groups (ACEI, BB, and MRA) were also homogenized. Finally, the propensity score was used in a 2:1 ratio because there were more patients without treatment than with it.

Statistical analysis was performed using R software, version 4.1.

This study was approved by the Ethics Committee of Hospital Fundación Alcorcón and was carried out in accordance with the ethical requirements of the Declaration of Helsinki. All included patients (or a representative relative) provided written consent, after being explained and understanding the above. There was no conflict of interest for the authors.

## 3. Results

Of the 750 patients (Table 1), 317 (42%) were men, the mean age was 84 years, and the mean Barthel index was 80 points. The most important comorbidities were arterial hypertension (679, 90.5%), atrial fibrillation (519, 69.2%), and hypercholesterolemia (481, 64.1%). Diabetes mellitus and chronic kidney disease were present in 46% and 47.5%, respectively. The other comorbidities are described in Table 1.

The most common etiology of decompensated heart failure was arterial hypertension (301, 40.1%), followed by valvular heart disease (184, 24.5%) and coronary ischemia (154, 20.5%). Regarding functional class according to the NYHA scale, 405 patients (54%) were classified as functional class II, and 279 (37.2%) were functional class III. The mean LVEF was 51%. Mean NT-proBNP was 5414 pg/mL, and CA-125 was 48 U/mL.

At discharge, most of the patients received furosemide (587, 78.3%) and beta-blockers (466, 62.1%). ACEIs/ARBs/ARNIs were administered in 52.4% (393) and MRAs in 35.6% (256). SGLT2is were the least-prescribed pharmacological group at discharge, in only 28% of patients, finding no differences between the drug options, (13.2% were on empagliflozin and 12.1% on dapagliflozin). Forty-two patients (5.6%) died during hospital admission; of these, cardiovascular death was noted in 71%. Over the next 12 months, 81 (10.8%) died, and 260 (35%) were readmitted to the hospital (Table 1).

Regarding those prescribed SGLT2is, men predominated (48.6% versus 39.8%, *p* = 0.029), they were younger (82 vs. 84, *p* = 0.002), with higher proportion of diabetes mellitus (63% vs. 39%, *p* < 0.001) and better functionality as measured by the Barthel Index (85 vs. 75, *p* = 0.036). 

In both sexes, arterial hypertension was the most frequent cause of HF decompensation (31.4% in the group with SGLT2is and 43.1% in those without it). In the group with SGLT2is at discharge, more coronary ischemia (25.2% vs. 18.7%) and toxic dilated cardiomyopathy (3.8% vs. 1.1%) were perceived to be responsible for the condition (*p* = 0.005). There were no statistically significant differences in terms of functional class, even though a lower LVEF was observed in patients treated with SGLT2is (48% vs. 52%, *p* < 0.001). Those who received the drug had a higher NT-proBNP at arrival (6418 pg/mL vs. 4900 pg/mL, *p* = 0.021), but they had similar CA-125 and creatinine values.

Regarding treatment, the rates of prescription of ACE inhibitors and ARBs were similar in both groups. However, it was observed that those who received SGLT2is at discharge were also on ARNI (20% in the treated group vs. 5.2% in the untreated group), BB (74.8% vs. 57.2%), and MRA (74.8% vs. 57.2%) treatment more frequently and concomitantly (*p* < 0.001). (Table 1).

**Figure 1:**
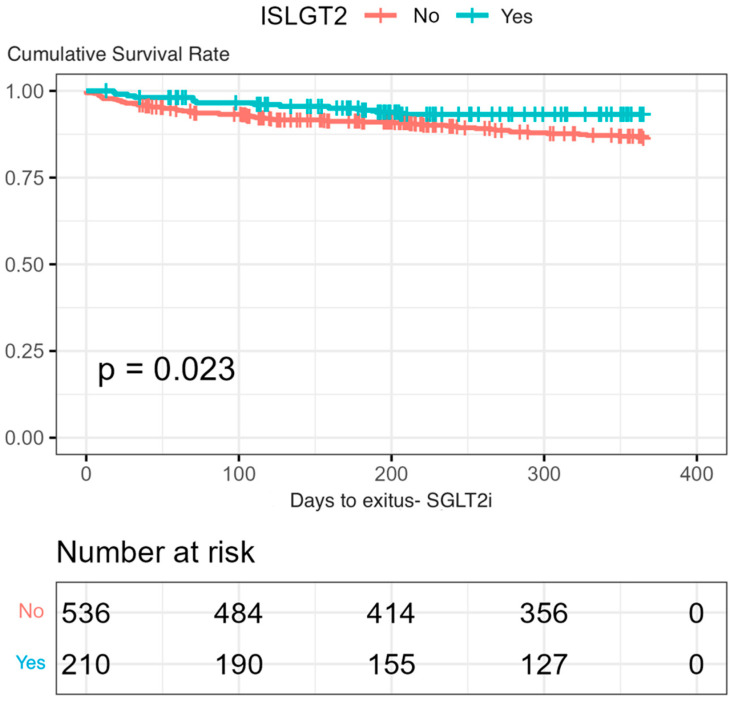
Kaplan–Meier curves of mortality analysis during the 12-month follow-up in the group treated with SGLT2is versus an untreated group (using log-rank test).

**Figure 2:**
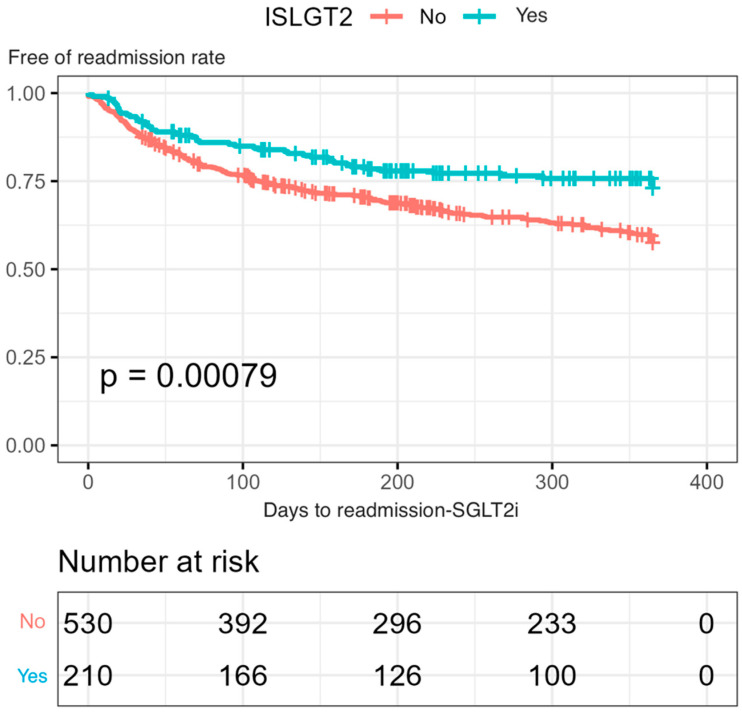
Kaplan–Meier curves of readmission analysis during a 12-month follow-up in the group treated with SGLT2is versus an untreated group (using log-rank test).

A statistically significant lower mortality was observed in the group treated with SGLT2is, both at admission (2.4% vs. 6.9%) (*p* = 0.017) and at 12 months’ follow-up (6.2% vs. 13%) (*p* = 0.023) (Figure 1). In addition, a lower readmission rate was observed during follow-up when SGLT2is were prescribed at discharge. Only 50 out of 260 readmissions (19.2%) were noted in the SGLT2is group (*p* < 0.001) (Figure 2).

Propensity score matching was performed. Only patients with an LVEF > 40% were included in this analysis in an effort to evaluate the effect of SGLT2is individually and eliminate any interactions with other treatments that demonstrate efficacy in heart failure with reduced ejection fraction. Drugs (ACEIs, BBs, and MRAs) in which statistically significant differences were observed in their administration between the two groups were also homogenized. Variables that impacted the severity of AHF were included (sex, age, systolic blood pressure (SBP), LVEF, creatinine, NT-proBNP, diabetes mellitus, and chronic kidney disease). The distribution of SBP, creatinine, and chronic kidney disease was homogeneous between the groups, so they were excluded from the analysis to avoid reducing the effectiveness of the test.

The results of the propensity score matching are shown in Table 2. In the SLGT2is group, we observed a tendency of a lower mortality rate during admission (1.5% vs. 5.2%) as well as during the 12-month follow-up (5.9% vs. 11.2%), although statistical significance was not reached (Table 2). However, statistically significant lower readmission rates continued to be observed in the group treated with SGLT2is (25% vs. 38.4%, *p* = 0.030). 

**Figure 3:**
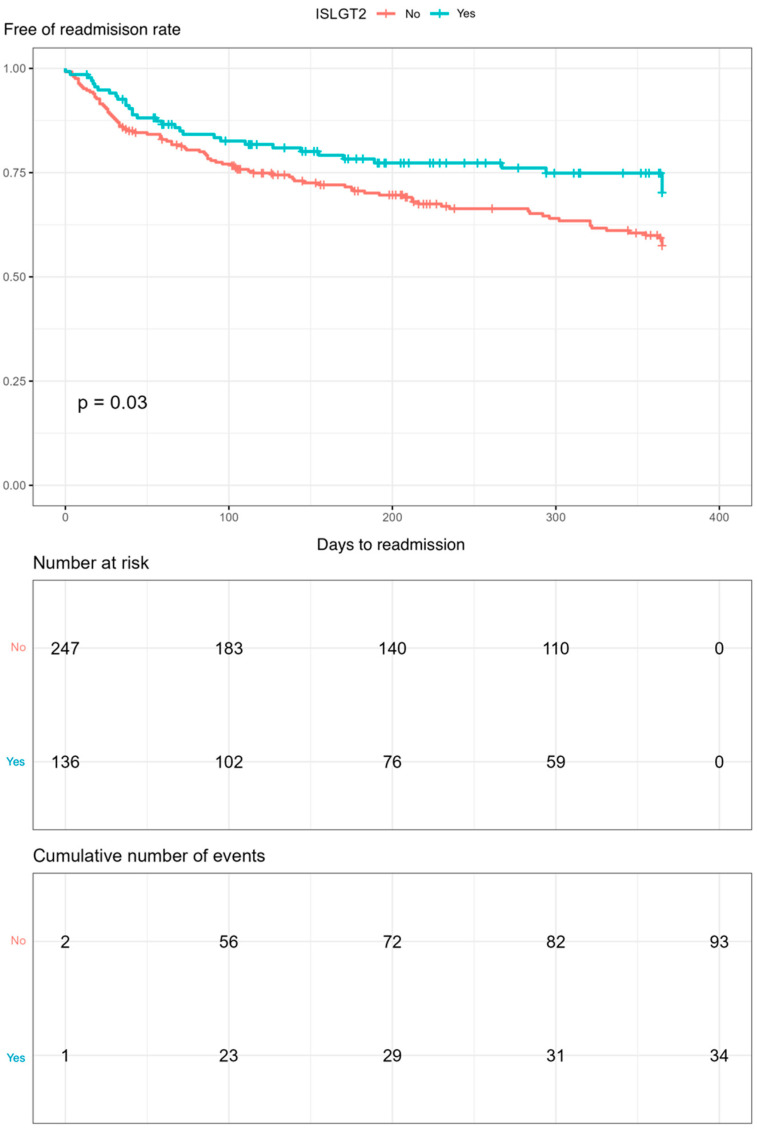
Kaplan–Meier curves of readmissions during the 12-month follow-up in the group treated with SGLT2is versus an untreated group (using log-rank test) after the propensity score matching.

**Figure 4:**
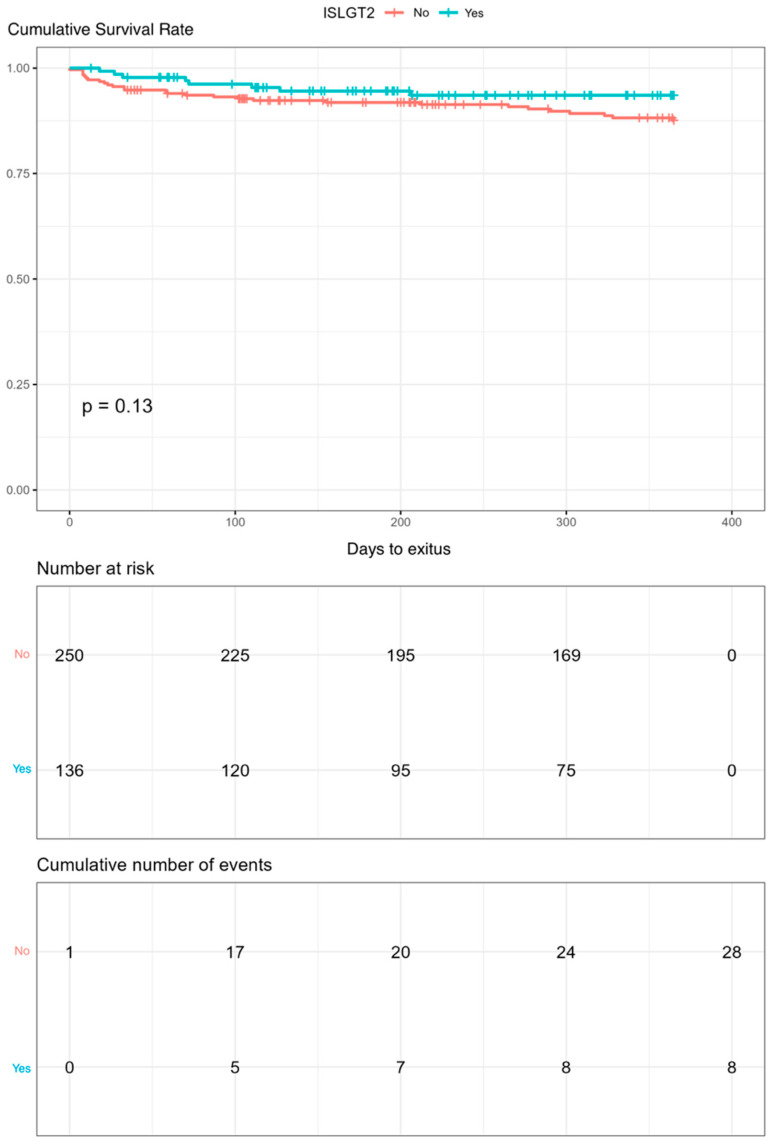
Kaplan–Meier curves of mortality during the 12-month follow-up in the group treated with SGLT2is versus the untreated group (using log-rank test) after the propensity score matching.

Kaplan–Meier curves for mortality and readmission at 12 months follow-up were drawn. SGLT2is use was found to reduce readmissions (*p* = 0.030, Figure 3). In addition, a tendency of decreased mortality rate during the 12-month follow-up was observed in the SGLT2is group, although statistical significance was not reached (Figure 4). 

According to the univariable Cox proportional hazards model, the use of SGLT2is was associated with fewer readmissions (HR 0.65, IC (0.44–0.96), *p* = 0.031) (Table 3). No significant differences were found in the univariate Cox proportional hazards model for death stratified by SGLT2is use (Table 4).

## 4. Discussion

In this study, SGLT2is prescription was associated with lower readmission rates during a 12-month follow-up in older patients with multiple pathologies admitted for acute heart failure. 

Previous studies evaluating the efficacy of SGLT2is [9,10] included a younger population (mean age of 71–72 years old) compared to our sample (84 years). This is one of the most important differences between this study and the previously published evidence. Frailty and comorbidity are more frequent in older adults. For this reason, we need drugs such as SGLT2is to act on several diseases and reduce polypharmacy and its associated risks. In addition, the analysis of EMPEROR-PRESERVED carried out according to age group (<65 years, between 65 and 74 years, 74 and 79 years, and >80 years) [20] highlighted that empagliflozin reduces the primary objective of death of cardiovascular origin and hospitalization for HF, as well as the first episode of HF and rehospitalization for HF, regardless of age, and without finding relevant differences in the rate of adverse effects between the different groups. Therefore, age should not be a reason to limit the use of these drugs.

Previous studies found that men made up the largest percentage of patients with HF [9,10], in contrast with this sample. This could be related to the fact that women have a longer life expectancy and, in general, have greater representation in the spectrum of HF with preserved LVEF. Given the age and the mean LVEF of the sample, women are more frequently affected. 

Regarding comorbidities, although the percentages of arterial hypertension and diabetes are similar to the rates observed in previous trials [9,10], the patients in this sample had a higher percentage of other associated comorbidities such as atrial fibrillation. In addition, previous articles included mostly patients with functional class II (almost 75%) [10] compared to the 54% with class II and 37.2% with class III seen in this study. DELIVER or EMPEROR-PRESERVED included patients with a mean LVEF of 54% because they analyzed SGLT2is use in HFpEF. This study aimed to analyze SGLT2is use regardless of LVEF, age, and comorbidities, so the mean LVEF observed was 51%, lower than that in the previous study [9,10]. 

The optimal timing for the use of SGLT2is in acute heart failure (AHF) is being evaluated in other studies. The STRONG-HF [15] trial proposed the implementation of triple therapy (ACEIs, ARMs, and BBs) at the end of the AHF episode, reaching maximum doses within two weeks of discharge. This intervention resulted in improved clinical outcomes and quality of life, and lower mortality from any cause and readmission rates due to HF after 180 days. 

In the STRONG-HF study, SGLT2is were not included because the start of the trial was prior to the recommendation of these drugs. Subsequently, the EMPULSE trial [18] was published, in which empagliflozin was started between one and five days after hospitalization for AHF, achieving significant clinical benefits in the treated group at 90 days (reductions in cardiovascular mortality and hospitalizations for HF, and improvement in quality of life). Furthermore, the results of DICTATE-AHF [19], which evaluated the initiation of dapagliflozin in AHF, were recently disclosed. In this study, the initiation of SGLT2is was carried out during hospitalization once the patient was stabilized. This suggests that starting these drugs during the episode of AHF could be beneficial. Although adverse effects were not recorded and this constitutes an important limitation, no higher readmission rates were observed in the treated group. Nevertheless, it is unknown what percentage of patients already received SGLT2is prior to admission and how many started during hospitalization, which is another limitation of this study. Patients in the treated group who died during admission could have been previously treated or could have started the drug during the study. This is an observational study with its inherent limitations, and a clinical trial should be conducted to analyze the early clinical benefit of SGLT2is therapy during admission.

The results on death and readmission are similar to those found in Spanish patients admitted for HF, at 9.2%, reaching 14.5% at one year, and a readmission rate of 32% [21]. However, these outcomes are significantly lower than the 28% cumulative mortality per year reported by the EVOLUTION-HF study [22], which could be related to differences in the population and access to the health system of the countries included.

Despite their recent recommendation, SGLT2is have strong evidence supporting their use. However, there is certain therapeutic inertia when using these drugs. In Spain, these agents are the last drugs to be introduced, and they are started during hospital admission in less than 50% of cases. In other countries, the prescription rate is also low, nearly 21% in an American study [23]. In addition, SGLT2is were more frequently prescribed in men with a lower mean age in both studies. Female sex, age over 75 years, and chronic kidney disease are limitations for prescribers when using this drug. SGLT2is implementation was also associated with increased concomitant intake of BBs, ARMs, and ACEIs/ARBs/ARNIs [23]. 

In the EVOLUTION-HF clinical trial [22], SGLT2i prescription rates were even lower, around 2 and 11%, depending on the country. Meanwhile, the use of BBs and ACEIs reached 60–80% and that of ARM was around 20–40%. This may be because SGLT2is are the last therapeutic group to have demonstrated their efficacy in this pathology, mainly in HFpEF. However, the use of SGLT2is has increased between two- and seven-fold, while the use of other established drugs has persisted at similar rates.

This study has some limitations: (1) the small sample size limits the extrapolation of the results to the population, (2) adverse effects secondary to the use of these drugs were not registered, (3) it is unknown what percentage of patients had received SGLT2is prior to admission and how many started during it. 

Furthermore, after carrying out the propensity score matching, the use of SGLT2is was associated with less readmission in those with an LVEF > 40%. Only these drugs have shown benefits in HFpEF, and it is easier to evaluate their independent effect than in HFrEF, where there could be interactions with other drugs with demonstrated benefits. Patients with a lower LVEF were excluded to avoid confusion from the concomitant intake of other treatments. We are aware that this was an observational study, with its accompanying limitations; to make firm statements a clinical trial should be carried out.

As its strengths, this study included older patients and those with comorbidities who represent the type of patients hospitalized in internal medicine wards.

## 5. Conclusions

SGLT2is use was associated with lower readmission rates at 12-month follow-up in patients with multiple pathologies diagnosed of acute heart failure. Therapeutic initiatives regarding treatment optimization are important.

## Figures and Tables

**Table 1:** Baseline characteristics of patients with heart failure and bivariate analysis based on SGLT2is treatment.

Variable	N = 750	No SGLT2is (N = 540)	SGLT2is (N = 210)	*p*
	**Epidemiological characteristics**			
**Sex**				*p* = 0.029
Women (n, %)	433 (58%)	325 (60.2%)	108 (51.4%)	
Men (n, %)	317 (42%)	215 (39.8%)	102 (48.6%)	
**Age (years) (mean, SD)**	84 (+/−9)	84 (+/−9)	82 (+/−2)	*p* = 0.002
**Weight (kg) (mean, SD)**	72 (+/−16)	71 (+/−16)	75 (+/−17)	*p* = 0.003
**Height (cm) (mean, SD)**	160 (+/−9)	160 (+/−9)	161 (+/−9)	*p* = 0.2
	**Comorbidities**			
**Diabetes mellitus (n, %)**	345 (46%)	213 (39.4%)	132 (62.9%)	*p* < 0.001
**Arterial hypertension (n, %)**	679 (90.5%)	484 (89.6%)	195 (92.9%)	*p* = 0.2
**Hypercholesterolemia (n, %)**	481 (64.1%)	347 (64.3%)	134 (63.9%)	*p* > 0.9
**Atrial fibrillation (n, %)**	519 (69.2%)	378 (70%)	141 (67.1%)	*p* = 0.5
**Chronic kidney disease (n, %)**	356 (47.5%)	253 (46.9%)	103 (49.1%)	*p* = 0.6
**Ischemic heart disease (n, %)**	237 (31.6%)	166 (30.7%)	71 (33.8%)	*p* = 0.4
**Chronic respiratory disease (asthma, COPD) (n, %)**	268 (35.7%)	189 (35%)	79 (37.6%)	*p* = 0.5
**Cerebrovascular disease (n, %)**	125 (16.7%)	90 (16.7%)	35 (16.7%)	*p* > 0.9
**Cognitive impairment (n, %)**	85 (11.3%)	67 (12.4%)	18 (8.6%)	*p* = 0.14
**Peripheral arterial disease (n, %)**	81 (10.9%)	53 (9.8%)	28 (13.3%)	*p* = 0.2
**Hematological or solid organ neoplasia (n, %)**	69 (9.2%)	50 (9.3%)	19 (9%)	*p* > 0.9
	**Functionality**			
**Barthel (median, IQR)**	80 (50, 95)	75 (50, 95)	85 (55, 100)	*p* = 0.036
	**Clinical Characteristics**			
**HF etiology**				*p* = 0.005
Hypertension (n, %)	301 (40.1%)	235 (43.1%)	66 (31.4%)	
Ischemia (n, %)	154 (20.5%)	101 (18.7%)	53 (25.2%)	
Toxic dilated (n, %)	14 (1.9%)	6 (1.1%)	8 (3.8%)	
Valvulopathy (n, %)	184 (24.5%)	131 (24.3%)	53 (25.2%)	
Amyloidosis (n, %)	21 (2.8%)	12 (2.2%)	9 (4.3%)	
Others (n, %)	75 (10%)	55 (10.2%)	20 (9.5%)	
Unknown (n, %)	1 (0.1%)	0	1 (0.5%)	
**Functional class (NYHA)**				*p* = 0.2
I (n, %)	39 (5.2%)	25 (4.6%)	14 (6.7%)	
II (n, %)	405 (54%)	300 (56%)	105 (50%)	
III (n, %)	279 (37.2%)	198 (36.7%)	81 (38.6%)	
IV (n, %)	24 (3.2%)	14 (2.6%)	10 (4.8%)	
Unknown (n, %)	3 (0.4%)	3 (0.6%)	0	
**LVEF (%) (mean, SD)**	51 (+/−12%)	52 (+/−11)	48 (+/−13)	*p* < 0.001
	**Analytical Characteristics**			
**NT-proBNP (pg/mL) (median, IQR)**	5414 (2670, 10,566)	4900 (2639, 9987)	6418 (3002, 12,225)	*p* = 0.021
**CA-125 (U/mL) (median, IQR)**	48 (22, 104)	49 (21, 107)	47 (23, 100)	*p* > 0.9
**Creatinine (mg/dL) (median, IQR)**	1 (1, 2)	1 (1, 2)	1 (1, 2)	*p* > 0.9
	**Treatment**			
**Diuretics (n, %)**				*p* = 0.004
Furosemide (n, %)	587 (78.3%)	415 (76.9%)	172 (81.9%)	
Thiazide (n, %)	8 (1.1%)	7 (1.3%)	1 (0.5%)	
Both (n, %)	84 (11.2%)	56 (10.4%)	28 (13.3%)	
**ACEI (n, %)**	158 (21.1%)	116 (21.5%)	42 (20%)	*p* = 0.7
**ARB (n, %)**	165 (22%)	124 (23%)	41 (19.5%)	*p* = 0.3
**ARNI (n, %)**	70 (9.3%)	28 (5.2%)	42 (20%)	*p* < 0.001
**BB (n, %)**	466 (62.1%)	309 (57.2%)	157 (74.8%)	*p* < 0.001
**MRA (n, %)**	267 (35.6%)	164 (30.4%)	103 (49.1%)	*p* < 0.001
**SGLT2is (n, %)**	210 (28%)	210 (28%)		
**Empagliflozin (n, %)**	99 (13.2%)	99 (13.2%)		
**Dapagliflozin (n, %)**	91 (12.1%)	91 (12.1%)		
**Unknown (n, %)**	20 (9.5%)	20 (9.5%)		
	**Prognostic variables**			
**MEESI-AHF 30 days (median, IQR)**	4 (3, 9)	4 (3, 9)	5 (3, 9)	*p* = 0.13
**MAGGIC 1 year (median, IQR)**	19 (13, 25)	19 (13, 27)	19 (13, 25)	*p* = 0.7
**MAGGIC 3 years (median, IQR)**	43 (32, 53)	43 (32, 56)	43 (32, 52)	*p* = 0.7
	**Surveillance**			
**Deaths during hospital admission (n, %)**	42 (5.6%)	37 (6.9%)	5 (2.4%)	*p* = 0.017
**Cardiovascular deaths (n, %)**	30 (71.4%)	26 (70.3%)	4 (80%)	*p* > 0.9
**Deaths during 12-month follow-up (n, %)**	81 (10.8%)	68 (13%)	13 (6.2%)	*p* = 0.023
**Readmission during 12-month follow-up (n, %)**	260 (35%)	210 (38.9%)	50 (23.8%)	*p* < 0.001

Legend: SD: standard deviation; COPD: chronic obstructive pulmonary disease; IQR: interquartile range; HF: heart failure; NYHA: New York Heart Association; LVEF: left ventricular ejection fraction; NT-proBNP: N-terminal pro-B-type natriuretic peptide; CA-125: carbohydrate antigen 125; ACEI: angiotensin-converting enzyme inhibitor; ARB: angiotensin receptor blocker; ARNI: angiotensin receptor-neprilysin inhibitor; BB: beta-blocker; MRA: mineralocorticoid receptor antagonist; SGLT2is: sodium-glucose cotransporter-2 (SGLT2) inhibitors; MEESI-AHF: multiple estimation of risk based on the emergency department Spanish score in patients with AHF; MAGGIC: Meta-analysis Global Group in Chronic Heart Failure.

**Table 2:** Propensity score matching ratio 2:1.

Variable	N = 386	No SGLT2is (N = 250)	SGLT2is (N = 136)	*p*
**Epidemiological characteristics**				
**Sex**				*p* = 0.6
Women (n, %)	225 (58.3%)	143 (57.2%)	82 (60.3%)	
Men (n, %)	161 (41.7%)	107 (42.8%)	54 (39.7%)	
**Age (years) (mean, SD)**	84 (+/−8)	84 (+/−9)	83 (+/−8)	*p* = 0.8
**Weight (kg) (mean, SD)**	74 (+/−17)	73 (+/−18)	75 (+/−17)	*p* = 0.2
**Height (cm) (mean, SD)**	160 (+/−9)	160 (+/−9)	160 (+/−9)	*p* > 0.9
**Comorbidities**				
**Diabetes mellitus (n, %)**	214 (55.8%)	134 (53.6%)	80 (58.8%)	*p* = 0.3
**Arterial hypertension (n, %)**	348 (90.2%)	224 (89.6%)	124 (91.2%)	*p* = 0.7
**Hypercholesterolemia (n, %)**	251 (65%)	166 (66.4%)	85 (62.5%)	*p* = 0.4
**Atrial fibrillation (n, %)**	275 (71.2%)	181 (72.4%)	94 (69.1%)	*p* = 0.6
**Chronic kidney disease (n, %)**	182 (47.2%)	119 (47.6%)	63 (46.3%)	*p* = 0.9
**Ischemic heart disease (n, %)**	107 (27.7%)	76 (30.4%)	31 (22.8%)	*p* = 0.11
**Chronic respiratory disease (asthma, COPD) (n, %)**	155 (40.2%)	102 (40.8%)	53 (20%)	*p* = 0.7
**Cerebrovascular disease (n, %)**	67 (17.4%)	41 (16.4%)	26 (19.1%)	*p* = 0.5
**Cognitive impairment (n, %)**	38 (9.8%)	25 (10%)	13 (9.6%)	*p* = 0.9
**Peripheral arterial disease (n, %)**	38 (9.8%)	26 (10.4%)	12 (8.8%)	*p* = 0.6
**Hematological or solid organ neoplasia (n, %)**	29 (7.5%)	19 (7.6%)	10 (7.4%)	*p* > 0.9
**Functionality**				
**Barthel (median, IQR)**	78 (50, 95)	75 (50, 95)	80 (50, 95)	*p* = 0.7
**Clinical characteristics**				
**SBP (mean, SD)**	139 (+/−26)	140 (+/−26)	138 (+/−25)	*p* = 0.7
**HF etiology**				*p* = 0.7
Hypertension (n, %)	160 (41.5%)	104 (41.6%)	56 (41.2%)	
Ischemia (n, %)	67 (17.4%)	46 (18.4%)	21 (15.4%)	
Toxic dilated (n, %)	3 (0.8%)	2 (0.8%)	1 (0.7%)	
Valvulopathy (n, %)	105 (27.2%)	68 (27.2%)	37 (27.2%)	
Amyloidosis (n, %)	10 (2.6%)	4 (1.6%)	6 (4.4%)	
Others (n, %)	41 (10.6%)	26 (10.4%)	15 (11%)	
**Functional class (NYHA)**				*p* = 0.2
I (n, %)	17 (4.4%)	7 (2.8%)	10 (7.4%)	
II (n, %)	220 (57%)	147 (58.8%)	73 (53.7%)	
III (n, %)	135 (35%)	88 (35.2%)	47 (34.6%)	
IV (n, %)	13 (3.4%)	7 (2.8%)	6 (4.4%)	
Unknown (n, %)	1 (0.3%)	1 (0.4%)		
**LVEF (%) (mean, SD)**	56 (+/−8)	56 (+/−7)	55 (+/−9)	*p* = 0.9
**HFpEF**				*p* = 0.3
40–49% (n, %)	66 (17.1%)	39 (15.6%)	27 (19.9%)	
50% or more (n, %)	320 (82.9%)	211 (84.4%)	109 (80.1%)	
**Analytical characteristics**				
**NT-proBNP (pg/mL) (median, IQR)**	4828 (2498, 8785)	4748 (2504, 8378)	5161 (2496, 9051)	*p* = 0.6
**Creatinine (mg/dL) (median, IQR)**	1 (1, 2)	1 (1, 2)	1 (1, 2)	*p* = 0.6
**CA-125 (U/mL) (median, IQR)**	47 (22, 94)	48 (22, 98)	45 (23, 83)	*p* = 0.7
**Treatment**				
**Furosemide (n, %)**	302 (78.2%)	195 (78%)	107 (78.7%)	*p* = 0.6
**ACEI (n, %)**	81 (21%)	51 (20.4%)	30 (22.1%)	*p* = 0.7
**ARB (n, %)**	96 (24.9%)	61 (24.4%)	35 (25.7%)	*p* = 0.8
**ARNI (n, %)**	10 (2.6%)	6 (2.4%)	4 (2.9%)	*p* = 0.7
**BB (n, %)**	251 (65%)	160 (64%)	91 (66.9%)	*p* = 0.6
**MRA (n, %)**	150 (38.9%)	96 (38.4%)	54 (39.7%)	*p* > 0.9
**Follow up**				
**Deaths during hospital admission (n, %)**	15 (3.9%)	13 (5.2%)	2 (1.5%)	*p* = 0.07
**Deaths during 12-month follow-up (n, %)**	36 (9.3%)	28 (11.2%)	8 (5.9%)	*p* = 0.13
**Readmissions during 12-month follow** **-up (n, %)**	130 (33.7%)	96 (38.4%)	34 (25%)	*p* = 0.03

Legend: SD: standard deviation; COPD: chronic obstructive pulmonary disease; IQR: interquartile range; HF: heart failure; NYHA: New York Heart Association; LVEF: left ventricular ejection fraction; HFpEF: heart failure with preserved ejection fraction; NT-proBNP: N-terminal pro-B-type natriuretic peptide; CA-125: carbohydrate antigen 125; ACEI: angiotensin-converting enzyme inhibitor; ARB: angiotensin receptor blocker; ARNI: angiotensin receptor-neprilysin inhibitor; BB: beta-blocker; MRA: mineralocorticoid receptor antagonist; SGLT2is: sodium-glucose cotransporter-2 (SGLT2) inhibitor.

**Table 3:** Univariate Cox proportional hazards model of readmissions by SGLT2is.

Characteristic	HR	95% CI	*p*
**SGLT2is**	0.65	0.44–0.96	*p* = 0.031

Legend: SGLT2is: sodium-glucose cotransporter-2 (SGLT2) inhibitors; HR: hazard ratio; CI: confidence interval.

**Table 4:** Univariate Cox proportional hazards model of death by SGLT2is.

Characteristic	HR	95% CI	*p*
**SGLT2is**	0.51	0.25–1.03	*p* = 0.062

Legend: SGLT2is: sodium-glucose cotransporter-2 (SGLT2) inhibitors; HR: hazard ratio; CI: confidence interval.

## Data Availability

The data presented in this study are available on request from the corresponding author. The data are not publicly available due to privacy restrictions.
